# High protein does not change autophagy in human PBMCs after 1 hour

**DOI:** 10.1172/jci.insight.188845

**Published:** 2025-07-15

**Authors:** Sanjna Singh, Célia Fourrier, Kathryn J. Hattersley, Leanne K. Hein, Jemima Gore, Alexis Martin, Linh V.P. Dang, Barbara King, Rachael A. Protzman, Paul J. Trim, Leonie K. Heilbronn, Julien Bensalem, Timothy J. Sargeant

**Affiliations:** 1 Lysosomal Health in Ageing, South Australian Health and Medical Research Institute (SAHMRI), Adelaide, South Australia, Australia.; 2 Faculty of Health and Medical Sciences, University of Adelaide, Adelaide, South Australia, Australia.; 3 Clinical Trials Platform and; 4 Proteomics, Metabolomics and MS-Imaging Core Facility, SAHMRI, Adelaide, South Australia, Australia.; 5 Adelaide Medical School, University of Adelaide, Adelaide, South Australia, Australia.; 6 Lifelong Health Theme, SAHMRI, Adelaide, South Australia, Australia.

**Keywords:** Cell biology, Metabolism, Autophagy

## To the Editor:

Autophagy is a catabolic quality control pathway triggered by stressors such as starvation or protein aggregates, mediating lysosomal turnover and restoring cellular homeostasis. Defects in autophagy are linked to neurodegeneration, atherosclerosis, and aging, and activating autophagy extends both lifespan and health span in preclinical models (1). Consequently, there is immense interest in stimulating autophagy in people. However, progress has been hampered by the lack of techniques to measure human autophagy. The gold standard involves blocking lysosomal degradation and quantifying the buildup of key autophagy protein LC3B-II as “flux,” i.e., material that would have been turned over in the ensuing time. While feasible in cultured human peripheral blood mononuclear cells (PBMCs) ex vivo (2), growth in culture alters their nutrient environment — a profound autophagy regulator — and does not accurately reflect physiological autophagy. Recently, we developed a test that measures PBMC flux in whole blood, preserving PBMC physiological environment (3), but so far it has been used only in observational studies.

**Figure 1:**
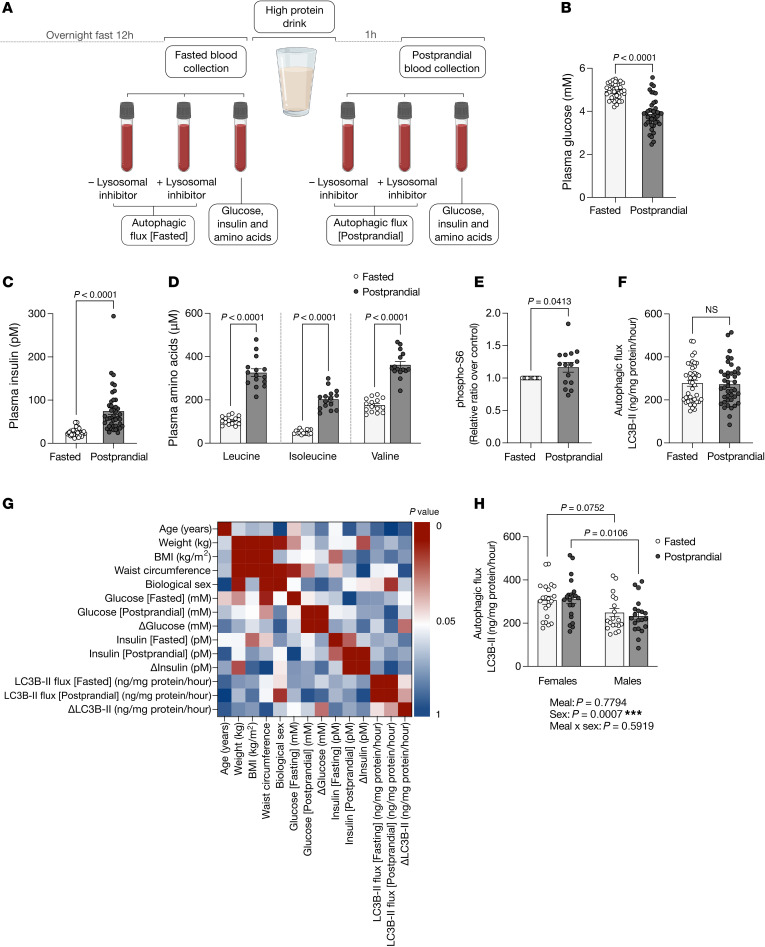
A high-protein meal does not alter autophagic flux in PBMCs. (**A**) Study design. (**B** and **C**) Fasted and postprandial plasma glucose (paired 2-tailed *t* test) and insulin (Wilcoxon’s test), *n* = 41; (**D**) branched-chain amino acids (paired 2-tailed *t* test), *n* = 15; (**E**) PBMC S6 phosphorylation (Wilcoxon’s test), *n* = 15; (**F**) autophagic flux (Wilcoxon’s test), *n* = 40; (**G**) Spearman’s correlations between blood measurements and baseline characteristics; and (**H**) 2-way ANOVA of autophagic flux.

Nutrient restriction is a universal activator of autophagy; conversely, high protein intake dampens autophagic flux in preclinical models. We conducted a single-arm pre-post study in 42 healthy individuals to assess whether 35 g protein could acutely modify autophagy in humans (Supplemental Methods; supplemental material available online with this article; https://doi.org/10.1172/jci.insight.188845DS1). We hypothesized that autophagic flux would be high in overnight-fasted individuals and suppressed following a protein-rich meal. Two blood samples were collected: after a 12-hour overnight fast and 1 hour after protein consumption (Figure 1A). Autophagic turnover was assessed by adding a lysosomal inhibitor to whole blood, with LC3B-II buildup (flux) measured via ELISA. The primary outcome was the change between fasted and postprandial autophagic flux (ΔLC3B-II). Effects on mTOR complex 1 (mTORC1) activity (an autophagy repressor) and plasma amino acid levels were measured in a separate cohort of 15 individuals.

Participant characteristics are presented in Supplemental Tables 1 and 2. Plasma glucose decreased and insulin increased 1 hour after protein consumption (Figure 1, B and C). Postprandial plasma amino acid levels, particularly branched-chain amino acids, rose significantly (Figure 1D and Supplemental Figure 1), alongside a modest increase in PBMC mTORC1 activity (Figure 1E). These data verify we were within the window to detect postprandial effects in blood. Strikingly, despite this, consumption of 35 g protein had no impact on PBMC autophagy: There were no differences between fasting and postprandial autophagic flux (Figure 1F). Spearman’s analyses revealed a correlation between flux and biological sex (Figure 1G). We stratified autophagy by sex and found that female individuals had higher flux (Figure 1H). A correlation was observed between change in autophagic flux and plasma glucose but was not pursued given the overall lack of flux change.

Here, we demonstrate that in contrast with animal models, which show autophagy is downregulated upon food intake (2, 4), autophagic flux in human PBMCs remains unchanged 1 hour after consumption of a protein-rich meal. Using nutrition to modulate autophagy is of interest to both the general public and scientific community — there are already products on the market claiming to activate autophagy in people via this pathway. Previous work has shown that LC3B-II decreased in human muscle 90 minutes after consuming a mixed macronutrient meal containing 22 g protein (5), but the study solely examined steady-state levels of LC3B-II, which did not provide information on autophagic turnover. Others have reported increased autophagic flux in PBMCs isolated after 24 hours of starvation in healthy human volunteers (2); however, measurement was performed ex vivo using nutrient-rich culture medium. This is suboptimal, since mTORC1 suppression in starved cells can rapidly reverse upon nutrient restimulation. This study is the first to our knowledge to examine autophagic flux in physiologically intact human tissue after an acute nutritional intervention. We show preliminary evidence suggesting that contrary to the widespread notion that fasting activates autophagy, autophagic flux in human PBMCs was unchanged between fasting and postprandial states, at least after 1 hour. However, we observed sexual dimorphism in autophagic flux, with females having higher levels than males.

Our work comes with some caveats. LC3B lipidation, our assay readout, also occurs during noncanonical autophagy, and its contribution to our measurements is unknown. While we observed expected changes in nutrient signaling 1 hour after consumption of a high-protein drink, this sampling time point may not have been optimal for detecting changes to flux. Further, tissue-specific autophagy responses are well documented in the literature (1). It is possible that our nutritional intervention changed flux in other tissues, such as liver or muscle, but not in PBMCs. This is an inherent limitation of human autophagy measurement because, at present, blood is the most accessible source of human tissue amenable to flux assays.

We have previously observed an age-associated increase in PBMC flux in an older, prediabetic cohort (6), as well as reduction in flux upon addition of exogenous insulin and leucine to blood (3). This suggests that, at least in some scenarios, our methodology is equipped to detect changes in autophagy.

In summary, we show that autophagic flux in human PBMCs remains unchanged 1 hour after protein ingestion. Importantly, this challenges the assumption that an acute nutritional stimulus can alter autophagy in people. Future work measuring PBMC flux in people at multiple time points will be key to understanding how it changes over time and whether it responds to acute nutritional interventions. Although augmenting this pathway is of immense clinical interest, our findings highlight potential discrepancies between preclinical and clinical modulation of autophagy, underscoring the need for further human autophagy research to answer the question, How can we boost autophagy in people?

## Supplementary Material

Supplemental data

Unedited blot and gel images

Supporting data values

## Version 1 07/15/2025

In-Press Preview

## Version 2 08/22/2025

Electronic publication

## References

[1] Aman Y (2021). Autophagy in healthy aging and disease. Nat Aging.

[2] Pietrocola F (2017). Metabolic effects of fasting on human and mouse blood in vivo. Autophagy.

[3] Bensalem J (2021). Measurement of autophagic flux in humans: an optimized method for blood samples. Autophagy.

[4] Seok S (2014). Transcriptional regulation of autophagy by an FXR-CREB axis. Nature.

[5] Morales-Scholz MG (2022). Muscle fiber type-specific autophagy responses following an overnight fast and mixed meal ingestion in human skeletal muscle. Am J Physiol Endocrinol Metab.

[6] Bensalem J (2023). Basal autophagic flux measured in blood correlates positively with age in adults at increased risk of type 2 diabetes. Geroscience.

