# Supplementary material for: High protein does not change autophagy in human PBMCs after 1 hour
Source: JCI Insight. 2025 Jul 15;10(16):e188845. doi: 10.1172/jci.insight.188845 (PMC12406713; doi:10.1172/jci.insight.188845)
Supplement: Supplemental data [file jciinsight-10-188845-s109.pdf]

SUPPLEMENTARY INFORMATION

Supplementary Table 1 Participant characteristics for autophagic flux analysis. Data are shown as mean ± SD for the full cohort (n=42) and separately for females (n=22) and males (n=20). Differences between sexes were analysed via Mann-Whitney test.

	N = 42	Females N = 22	Males N = 20	*p-value*
Age (years)	32.5 ± 7.1	32.6 ± 7.2	32.5 ± 7.1	0.935
Females, n (%)	22 (52.4)			
Body weight (kg)	75.1 ± 11.2	70.2 ± 10.8	80.5 ± 9.2	0.002
Body mass index (kg/m^2)	25.1 ± 2.7	24.7 ± 2.9	25.6 ± 2.5	0.435
Waist circumference (cm)	80.2 ± 8.3	76.3 ± 7.8	84.5 ± 6.8	0.001
Glucose [Fasting] (mM) (N = 40)*	4.9 ± 0.3	4.8 ± 0.3	5.0 ± 0.3	0.072
Glucose [Postprandial] (mM)	3.9 ± 0.7	3.8 ± 0.6	4.0 ± 0.8	0.676
ΔGlucose (mM) (N = 40)*	-1.1 ± 0.7	-1.1 ± 0.6	-1.1 ± 0.7	0.943
Insulin [Fasting] (pM) (N = 40)*	25.5 ± 9.1	26.0 ± 8.8	25.0 ± 9.5	0.600
Insulin [Postprandial] (pM)	74.4 ± 50.0	81.2 ± 54.4	66.8 ± 44.7	0.090
ΔInsulin (pM) (N = 40)*	47.4 ± 46.5	55.2 ± 51.6	38.3 ± 39.2	0.048
LC3B-II flux [Fasting] (ng/mg protein/hr) (N = 41)#	276.9 ± 89.7	303.8 ± 86.9	248.7 ± 85.8	0.045
LC3B-II flux [Postprandial] (ng/mg protein/hr) (N = 41)#	273.8 ± 94.2	312.6 ± 92.3	233.0 ± 79.4	0.008
ΔLC3B-II flux (ng/mg protein/hr) (N = 40)#	-2.3 ± 69.8	11.2 ± 84.1	-15.7 ± 50.5	0.327

* Fasted plasma glucose and insulin measurements missing for 1 participant.

# Autophagy measurement (either fasting or postprandial) excluded for 2 participants due to coefficient of variation (CV) exceeding 15%.

Supplementary Table 2 Participant characteristics for nutrient signalling analysis. Data are shown as mean ± SD Data for the full cohort (n=15) and separately for females (n=8) and males (n=7). Differences between sexes were analysed via Mann-Whitney test.

	**N = 15**	**Females N = 8**	**Males N = 7**	***p-value***
Age (years)	33.6 ± 10.4	34.0 ± 12.1	33.1 ± 9.1	0.979
Females, n (%)	8 (53.3)			
Body weight (kg)	72.2 ± 13.0	65.0 ± 11.0	80.3 ± 10.4	0.014
Body mass index (kg/m^2)	23.6 ± 2.8	23.0 ± 3.0	24.2 ± 2.7	0.463
Waist circumference (cm)	78.0 ± 8.9	72.6 ± 6.0	84.2 ± 7.8	0.014
Insulin [Fasting] (pM)	23.7 ± 9.5	25.4 ± 12.6	21.7 ± 3.9	0.842
Insulin [Postprandial] (pM)	82.4 ± 43.7	97.2 ± 41.4	65.4 ± 42.7	0.026
ΔInsulin (pM)	58.7 ± 37.2	71.8 ± 30.7	43.7 ± 40.5	0.021

Supplementary Figure 1 Fasted and postprandial plasma amino acids. (A-Q) Plasma levels of amino acids increase 1 h post-consumption of a high protein drink (paired t-test, n=15).

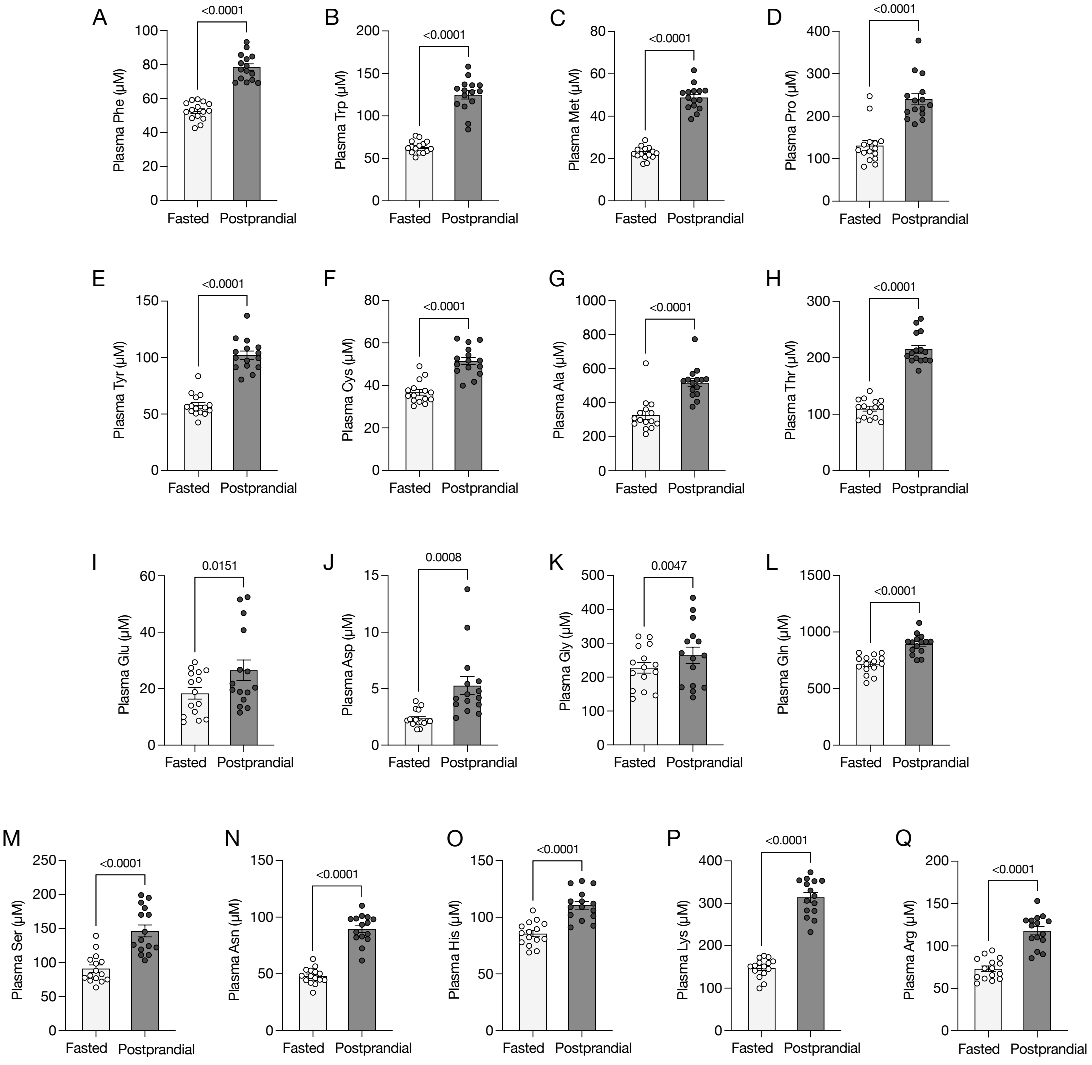

Supplementary Figure 2 Saponin extraction of non-lipidated LC3B. Three individual vacutainers containing blood were taken from one participant and processed in parallel. Cell pellets were saponin washed and both the pellet (P) and the saponin wash supernatant (S; containing soluble cytosolic proteins) analysed via western blot, showing that LC3B-II is detected in the pellet, and LC3B-I is found in the supernatant.

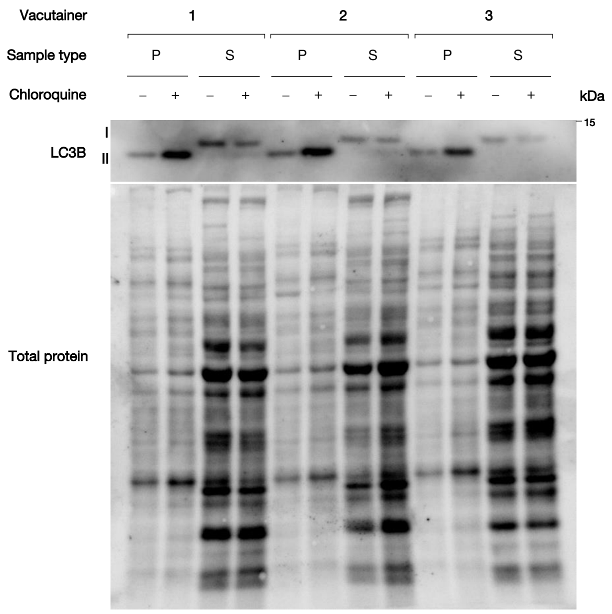

METHODS

Study design and setting

In this report, we present results from 42 participants enrolled in a prespecified, pre-post single arm study for measurement of autophagic flux between March and September 2022, and a smaller cohort of 15 participants between February 2025 and March 2025 for measurement of nutrient signalling (Australian New Zealand Clinical Trials Registry ACTRN12621001029886). The study was performed at the South Australian Health and Medical Research Institute (SAHMRI) by researchers from the University of Adelaide and SAHMRI. The inclusion criteria were: aged 20-50 years with a BMI of 18.5-29.9 kg/m^2. Key exclusion criteria were: co-morbidities likely to affect lysosomal function (e.g. cancer, cardiovascular conditions, neurological disorders), alcohol and/or substance use disorders (self-reported), and taking medications capable of altering autophagic activity or metabolism (e.g. blood glucose lowering medications, anti-inflammatories). Participants consuming common supplements such as iron, vitamin C and D, and fish oil were not excluded. The detailed study protocol with the complete list of inclusion and exclusion criteria has been published previously (1).

No randomisation was required as each participant served as their own control; all participants provided a pre- and post-intervention blood sample.

Sex as a biological variable

Both male and female participants were included in this study, and the effect of sex on autophagy was assessed.

Intervention

Participants were instructed to fast overnight for 12h prior to the study visit. Anthropomorphic data were collected as previously described (1). A fasting blood sample (20 mL) was drawn. Immediately following blood collection, participants were asked to consume a standardised whey protein drink (30 g unflavoured whey isolate powder diluted in 250 mL skim milk: 842 kJ, Protein 35.25 g, Fats 0.65 g, Carbohydrates 12.2 g) within 5 min. This high-protein, low-carbohydrate drink was expected to increase plasma insulin and reduce plasma glucose, as previously reported (2) – thus inhibiting autophagy via both the insulin and glucose axis. Participants were instructed to remain sedentary for 1h post-consumption of the high-protein drink, after which a second, postprandial blood sample (30 mL) was collected.

Outcomes

The primary outcome of this study was to measure change in autophagic flux after consumption of a protein-rich drink. Secondary outcomes included assessment of changes in nutrient-related signalling such as plasma glucose, amino acids and insulin post-consumption of a high-protein drink.

PBMC isolation

Blood samples were incubated with a lysosomal inhibitor to measure autophagic flux, as described below, prior to PBMC isolation. All PBMC extraction steps were performed on ice (or at 4°C during centrifugation) with cold buffers to inhibit further vesicle trafficking. Blood underwent a 1:1 dilution in Dulbecco's Phosphate Buffered

Saline (DPBS; Thermo Fisher Scientific, 14190136). PBMCs were isolated by underlaying the blood-DPBS mixture with Lymphoprep (Stemcell Technologies, 07811) and centrifuging for 30 min at 800 x g, with deceleration set to 1. The PBMC layer was collected, diluted in DPBS and pelleted via centrifugation at 600 x g for 10 min. The PBMC pellet was resuspended in 1 mL red blood cell lysis buffer (BD Biosciences, 555899) and incubated on ice for 2 min to remove residual red blood cells. PBMCs were washed twice using DPBS and centrifuged at 600 x g for 10 min. Pellets were resuspended in 1 mL DPBS, transferred to microcentrifuge tubes, and spun at 2,000 x g for 10 min. The supernatant was discarded and PBMC pellets snap-frozen on dry ice and stored at -80°C for biochemical analysis.

Autophagic flux measurement

Autophagic flux was assessed for both fasted and postprandial blood samples immediately following collection, using methodology developed by our group (3,4). Briefly, 6 mL of blood was divided into 2 tubes – one served as the control, while the other was treated with 150 μM of the lysosomal inhibitor chloroquine (Sigma Aldrich, C6628). Both tubes were incubated for 1 h at 37°C with rotation. All subsequent steps were performed on ice (or at 4°C during centrifugation) with cold buffers to inhibit further vesicle trafficking. PBMCs were processed as described above, with cell pellets snap-frozen on dry ice and stored at -80°C for biochemical analysis.

PBMC pellets were thawed on ice and resuspended in 0.05% saponin (Sigma Aldrich, SAE0073) in DPBS containing protease (Sigma Aldrich, 4693132001) and phosphatase inhibitors (2.5 mM sodium pyrophosphate, 1 mM sodium orthovanadate,

1 mM β-glycerophosphate) for 5 min with gentle rocking. This saponin wash served as a weak permeabilizing agent, removing the cytosolic pool of LC3B-I, without affecting membrane associated LC3B-II, which is what accumulates upon lysosomal inhibition *i.e.* the species used to quantify autophagic flux (Supplementary Figure 2). PBMCs were washed in DPBS, re-pelleted and resuspended in cell extraction buffer (Cell Signalling Technology, 35172) containing protease inhibitors. Cell suspensions were sonicated on ice (2x 20 s bursts) and clarified at 16,000 x g for 5 min at 4ºC. The supernatant was collected, and protein concentration determined via a micro-BCA protein assay kit (Thermo Fisher, 23235).

LC3B-II concentration was measured by loading 5 *µ*g clarified cell lysate per well in triplicate on a FastScan Total LC3B ELISA kit (Cell Signalling Technology, 35172). A standard curve was prepared using 0 – 4 ng/well recombinant human LC3B (Abcam, ab103506). ELISA plates were prepared as per manufacturer's instructions, and the absorbance measured at 450 nm using Glomax plate reader (Promega). LC3B-II protein concentrations of each sample were interpolated from the standard curve and reported as ng LC3B-II/mg of total protein/hour. Due to a technical issue with the standard curve on one plate, the LC3B-II concentrations for that plate were interpolated using the average of standard curves generated from the other plates within the same batch. Autophagic flux was calculated as:

ΔLC3B-II (ng LC3B-II/mg protein/hour) = LC3B-II [Chloroquine] – LC3B-II [Control]

Autophagic flux was measured in 84 samples (fasting and postprandial samples for n = 42 participants). Two samples (from different participants) were excluded due to CV between triplicate wells being > 15%. Therefore, final autophagic flux analysis was performed on 82 samples from 42 participants.

Plasma amino acid measurement

Plasma amino acids were quantified in 30 samples (fasted and postprandial samples from 15 participants) using liquid chromatography-tandem mass spectrometry (LC-MS/MS). Samples were thawed on ice, diluted 1:10 in water, and mixed with 10 μL of 10 μM stable isotope-labelled amino acid internal standard mixture (Sigma-Aldrich, 909653). Proteins were precipitated with methanol, centrifuged for 5 min at 16,000 x g and supernatants transferred to a 96-well plate for analysis. LC-MS/MS analysis (scheduled MRM) was performed using an API 5500 QqQ mass spectrometer (AB/Sciex) coupled to an Acquity UPLC system (Waters) equipped with an Acquity premier BEH Amide column (2.1 × 100 mm, 1.7 μm; Waters). Amino acid quantification was performed using internal standard calibration, and a physiological amino acid standard (Sigma, A9906) was included as a quality control reference.

Plasma metabolic measurements

Participant blood was collected in 9 mL EDTA or 2 mL sodium fluoride/potassium oxalate tubes for insulin and glucose measurements, respectively. For glucose measurements, blood was centrifuged at 3000 x g for 15 min at room temperature, with the plasma layer aspirated and stored at -80°C. Plasma glucose was measured using the hexokinase method (Cobas Integra 400 plus, Roche). For plasma insulin

measurements, blood was centrifuged at 1500 x g for 10 min at room temperature, after which the plasma layer was aspirated and stored at -80ºC. Plasma insulin was assessed using a commercially available ELISA kit (Mercodia, 10-1113-01).

S6 phosphorylation measurement in PBMCs

PBMC pellets were thawed on ice and resuspended in lysis buffer (Cell Signalling Technology, 9803) containing protease inhibitors. Cell suspensions were sonicated on ice (2x 10 s bursts) and clarified at 16,000 x g for 5 min at 4ºC. The supernatant was collected, and protein concentration determined using a micro-BCA protein assay kit (Thermo Fisher, 23235). S6 phosphorylation was measured by loading 12 μg clarified cell lysate per well in duplicate on a FastScan phospho-S6 (Ser240/244) ELISA kit (Cell Signalling Technology, 13911). ELISA plates were prepared as per manufacturer's instructions, and the absorbance measured at 450 nm using a Glomax plate reader (Promega).

Western blot

Equal volumes of saponin-washed PBMC supernatants or pellets were electrophoresed through 4-12% BOLT™ (Thermo Fisher Scientific, NW04122) gels at 125V for 1-1.5 h, and transferred on PVDF membranes at 35V for 70 min. Membranes were incubated for 1 h at room temperature in blocking buffer comprising 5% skim milk in Tris-buffered saline containing 0.1% Tween and incubated with primary antibody (Novus Biologicals rabbit anti-LC3B 1:1000, NB100-2220) overnight at 4ºC. Where indicated, total protein staining was performed before the blocking step, as per manufacturer's instructions (Invitrogen, A44449). The following day, membranes were

washed and incubated with HRP-conjugated secondary antibodies (Merck; AP307P 1:10 0000). Membranes were washed, developed with chemiluminescent substrates (Thermo Fisher, 34096) and imaged on a Fujifilm LAS4000.

Statistics

Statistical analysis was performed using GraphPad Prism Version 10.1.1 for MacOS. Data distribution was assessed using Shapiro-Wilk test. Missing values were not imputed. Depending on data distribution, either a two-tailed paired sample t-test or Wilcoxon signed rank test was used to test for differences between fasted and postprandial measurements. Spearman's correlation and two-way ANOVA were used for exploratory analyses. Error bars in all figures represent SEM. A p value less than 0.05 was considered statistically significant. Details for the statistical tests used are provided in the figure legend.

Study approval

The study protocol was approved by the University of Adelaide Human Research Ethics Committee (H-2021-024). Written informed consent was obtained from all participants.

Data availability

Values for all data points used in Figure 1 and Supplementary Figure 1 can be found in the Supporting Data Values file. Unprocessed blots for Supplementary Figure 2 are provided 'Full unedited blot for Supplementary Figure 2'. No custom code was used in

this study. Additional raw data generated during the study but not included in the manuscript are available from the corresponding author upon reasonable request.

ACKNOWLEDGEMENTS

We acknowledge Xiao Tong Teong for help with glucose measurement, and Kristin Mellander Smith for help with participant recruitment and visits. Parts of Figure 1A were created in BioRender: Sargeant, T. (2025) https://BioRender.com/oixeejl

REFERENCES

1. Bensalem J, et al. The Break-Fast study protocol: a single arm pre-post study to measure the effect of a protein-rich breakfast on autophagic flux in fasting healthy individuals. *BMC Nutr*. Nov 1 2022;8(1):120.

2. Hutchison AT, et al. Acute load-dependent effects of oral whey protein on gastric emptying, gut hormone release, glycemia, appetite, and energy intake in healthy men. *Am J Clin Nutr*. Dec 2015;102(6):1574-84.

3. Bensalem J, et al. Measurement of autophagic flux in humans: an optimized method for blood samples. *Autophagy*. Oct 2021;17(10):3238-3255.

4. Bensalem J, et al. Basal autophagic flux measured in blood correlates positively with age in adults at increased risk of type 2 diabetes. *Geroscience*. Dec 2023;45(6):3549-3560.
