# Supplementary figures and images for: High protein does not change autophagy in human PBMCs after 1 hour
Source: JCI Insight. 2025 Jul 15;10(16):e188845. doi: 10.1172/jci.insight.188845 (PMC12406713; doi:10.1172/jci.insight.188845)

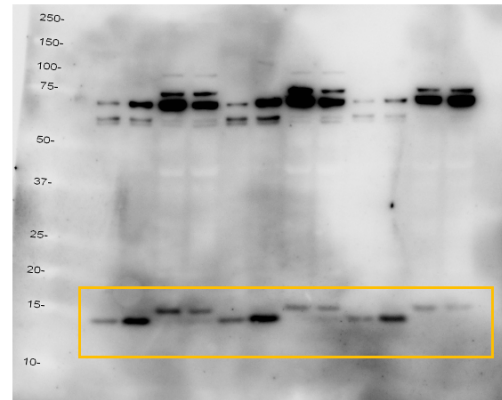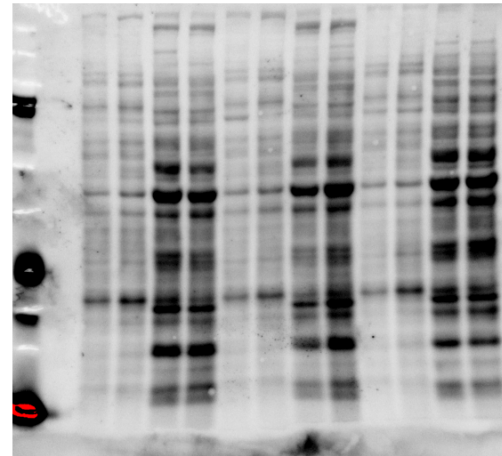

Supplement: Unedited blot and gel images [file jciinsight-10-188845-s110.pdf]
